# P-1554. Impact of High Predicted Risk of Antimicrobial Resistance on Empiric Treatment Failure in US Females with Uncomplicated Urinary Tract Infections

**DOI:** 10.1093/ofid/ofae631.1721

**Published:** 2025-01-29

**Authors:** Debra L Fromer, Meghan Luck, Malena Mahendran, Rose Chang, Megan Pinaire, Dar Alon, Mei Sheng Duh, Madison T Preib, Jeffrey J Ellis

**Affiliations:** Hackensack University Medical Center / Hackensack Meridian School of Medicine, Hackensack, NJ; GSK; Analysis Group, Inc., Boston, Massachusetts; Analysis Group, Inc., Boston, Massachusetts; Analysis Group, Inc., Boston, Massachusetts; Analysis Group, Inc., Boston, Massachusetts; Analysis Group, Inc., Boston, Massachusetts; GSK; GSK

## Abstract

**Background:**

Antibiotic (ABX) treatment of uncomplicated urinary tract infection (uUTI) is often prescribed empirically which may lead to treatment failure (TF) and antimicrobial resistance (AMR). Suspected AMR may be assessed using a published AMR risk categorization framework. This study aimed to investigate the impact of high predicted risk of AMR on TF in female outpatients with uUTI.

**Figure:**
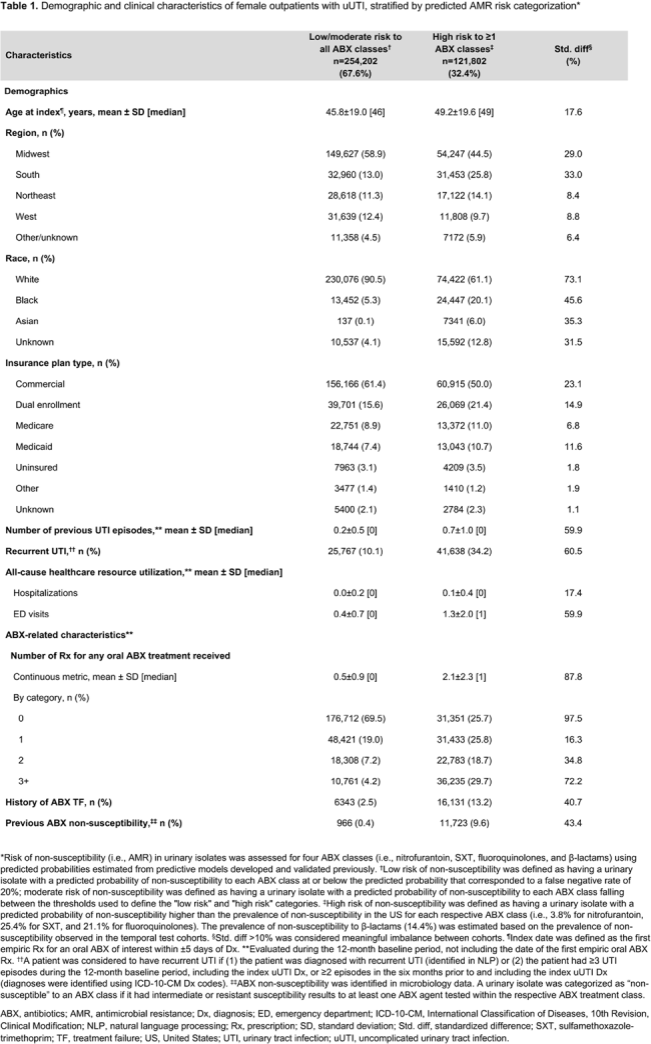

**Methods:**

Female outpatients aged ≥ 12 years with a uUTI diagnosis (Dx) between Jan 2018 and Sep 2022 were assessed retrospectively using Optum’s de-identified Electronic Health Record dataset. TF was defined as having a second oral ABX prescription (Rx), intravenous ABX, or emergency department/inpatient stay with a primary Dx of UTI ≤ 28 days after the first empiric oral ABX Rx for the uUTI Dx. Four validated AMR risk predictive models categorized patients as low or moderate (L/M) or high risk of isolate non-susceptibility to each of nitrofurantoin, sulfamethoxazole-trimethoprim, fluoroquinolones, and β-lactams, separately. Patients predicted to have high risk of isolate non-susceptibility to ≥ 1 ABX class were categorized as high AMR risk. Risk ratios (RRs) with 95% confidence intervals (CIs) were used to compare TF in patients with L/M AMR risk vs high AMR risk and the effect of recurrent uUTI history on TF in high AMR risk patients was explored.

**Figure:**
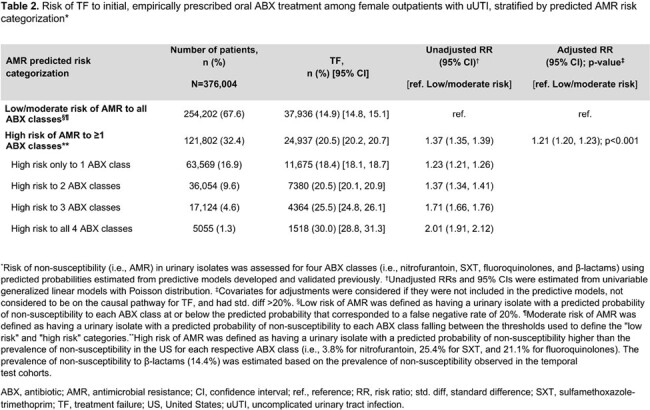

**Results:**

Of 376,004 female outpatients with uUTI, 121,802 (32.4%) were categorized as high AMR risk (**Table 1**). Risk of TF was higher in patients with high AMR risk vs L/M AMR risk (21% vs 15%, adjusted RR [95% CI] = 1.21 [1.20–1.23]; p< 0.001; **Table 2**). TF risk increased with number of predicted high risk ABX classes, as patients with 1, 2, 3, and 4 predicted high risk ABX classes had unadjusted TF RRs of 1.23, 1.37, 1.71, and 2.01, respectively, vs those with L/M AMR risk (all p< 0.001; **Table 2**). TF rates were higher in patients with high AMR risk and recurrent UTI history vs L/M AMR risk and non-recurrent UTI (23.1% vs 14.6%, unadjusted RR [95% CI] = 1.58 [1.55–1.62]; **Table 3**).

**Figure:**
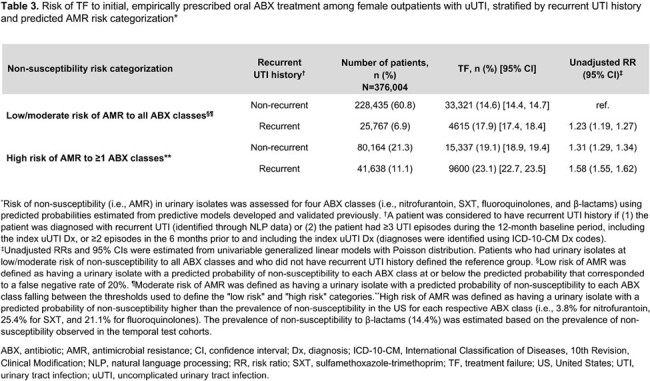

**Conclusion:**

Female uUTI outpatients with high predicted AMR risk to ≥ 1 oral ABX had a significantly greater risk of TF vs those with L/M predicted risk of AMR. These findings underscore the clinical importance of suspected AMR and history of UTI recurrence in empiric treatment decision-making for uUTI.

**Funding:**

GSK study 219500

**Disclosures:**

**Debra L. Fromer, MD**, GSK: Advisor/Consultant|Johnson & Johnson/Janssen Pharmaceuticals: Advisor/Consultant **Meghan Luck, PharmD, BCPS**, GSK: Employee|GSK: Stocks/Bonds (Private Company) **Malena Mahendran, MS**, Analysis Group, Inc.: Employee of Analysis Group, Inc., a consulting company that received funding from GSK to conduct this study **Rose Chang, ScD**, Analysis Group, Inc.: Employee of Analysis Group, Inc., a consulting company that received funding from GSK to conduct this study **Megan Pinaire, MPH**, Analysis Group, Inc.: Employee of Analysis Group, Inc., a consulting company that received funding from GSK to conduct this study **Dar Alon, MS**, Analysis Group, Inc.: Employee of Analysis Group, Inc., a consulting company that received funding from GSK to conduct this study **Mei Sheng Duh, MPH, ScD**, Analysis Group, Inc.: Employee of Analysis Group, Inc., a consulting company that received funding from GSK to conduct this study **Madison T. Preib, MPH**, GSK: Employee|GSK: Stocks/Bonds (Public Company) **Jeffrey J. Ellis, PharmD, MS**, GSK: Employee|GSK: Stocks/Bonds (Public Company)

